# Clinical value of second opinions in oncology: A retrospective review of changes in diagnosis and treatment recommendations

**DOI:** 10.1002/cam4.5598

**Published:** 2023-02-03

**Authors:** Allison Lipitz‐Snyderman, Susan Chimonas, Sham Mailankody, Michelle Kim, Nicholas Silva, Anuja Kriplani, Leonard B. Saltz, Smita Sihag, Carlyn Rose Tan, Maria Widmar, Marjorie Zauderer, Saul Weingart, Wendy Perchick, Benjamin R. Roman

**Affiliations:** ^1^ Department of Epidemiology and Biostatistics Memorial Sloan Kettering Cancer Center New York New York USA; ^2^ Department of Medicine Memorial Sloan Kettering Cancer Center New York New York USA; ^3^ Strategy and Innovation Memorial Sloan Kettering Cancer Center New York New York USA; ^4^ Department of Surgery Memorial Sloan Kettering Cancer Center New York New York USA; ^5^ Rhode Island Hospital and Hasbro Children's Hospital Providence Rhode Island USA

**Keywords:** diagnostic change, morbidity and prognosis, oncology, second opinion, treatment change

## Abstract

**Background:**

Data on the clinical value of second opinions in oncology are limited. We examined diagnostic and treatment changes resulting from second opinions and the expected impact on morbidity and prognosis.

**Methods:**

This retrospective cohort study included patients presenting in 2018 to a high‐volume cancer center for second opinions about newly diagnosed colorectal, head and neck, lung, and myeloma cancers or abnormal results. Two sub‐specialty physicians from each cancer type reviewed 30 medical records (120 total) using a process and detailed data collection guide meant to mitigate institutional bias. The primary outcome measure was the rate of treatment changes that were “clinically meaningful”, i.e., expected to impact morbidity and/or prognosis. Among those with treatment changes, another outcome measure was the rate of clinically meaningful diagnostic changes that led to treatment change.

**Results:**

Of 120 cases, forty‐two had clinically meaningful changes in treatment with positive expected outcomes (7 colorectal, 17 head and neck, 11 lung, 7 myeloma; 23–57%). Two patients had negative expected outcomes from having sought a second opinion, with worse short‐term morbidity and unchanged long‐term morbidity and prognosis. All those with positive expected outcomes had improved expected morbidity (short‐ and/or long‐term); 11 (0–23%) also had improved expected prognosis. Nine involved a shift from treatment to observation; 21 involved eliminating or reducing the extent of surgery, compared to 6 adding surgery or increasing its extent. Of the 42 with treatment changes, 13 were due to clinically meaningful diagnostic changes (1 colorectal, 5 head and neck, 3 lung, 4 myeloma; 3%–17%) .

**Conclusions:**

Second‐opinion consultations sometimes add clinical value by improving expected prognoses; more often, they offer treatment de‐escalations, with corresponding reductions in expected short‐ and/or long‐term morbidity. Future research could identify subgroups of patients most likely to benefit from second opinions.

## Abstract 2

In a retrospective review of the clinical value of second opinions in oncology, between 23% and 57% of patients across four disease types had significant changes in management from the second opinion expected to impact outcomes of morbidity and prognosis. De‐escalations in care, including some to no treatment needed, were common, with resulting expected reductions in morbidity.

## PRECIS

In a retrospective review of the clinical value of second opinions in oncology, between 23% and 57% of patients across four disease types had expected improvements in morbidity and prognosis due to a change in treatment from the second opinion. De‐escalations in care, including some to no treatment needed, were common, with resulting expected reductions in morbidity.

## 1 INTRODUCTION

Second opinions in oncology may be a mechanism for improving outcomes by providing a critical check on the accuracy of patients' diagnoses and appropriateness of their treatment plans.^1^, ^2^ This belief is supported by studies documenting frequent diagnostic or treatment changes from second opinions.^3^, ^4^, ^5^, ^6^ The purported value is such that many medical centers advertise their second opinion services in oncology, and online digital health companies facilitate such consultations.^3^

Yet data on the clinical value of second‐opinions are limited. Studies of diagnostic or treatment changes have mostly been limited to single diseases or patient populations (e.g., breast cancer),^4^, ^7^, ^8^, ^9^ or single diagnostic modalities (e.g., pathology review).^10^, ^11^ Most importantly, there is limited information regarding the impact of second opinions on the clinically meaningful outcomes of prognosis and morbidity. This information is crucial to identifying where and by what mechanisms added value may accrue.

To address these critical gaps, we performed a retrospective review of second opinion consultations at a high‐volume cancer center with subspeciality expertise. Spanning 4 disease types—colorectal cancers, head and neck cancers, lung cancers, and multiple myeloma—we examined the entire specialty second opinion process from diagnostic review to treatment planning. Our goals were first, to identify the specific mechanisms by which second opinions add value, be it by catching diagnostic or treatment errors, offering additional tests or treatment options, or refining patients' care plans and treatment modalities; and second, to determine the expected impacts of these changes on morbidity and prognosis. Because of several limitations including the risk of institutional bias among our reviewers, we aimed not to firmly establish rates of change from second opinion consultations but, rather, to assess subgroup heterogeneity and examine in‐depth the various ways in which second opinions in oncology can be clinically valuable.

## 2 METHODS

### 2.1 Study design

We conducted physician‐led retrospective reviews of medical records for cases where a second opinion was sought for a new diagnosis of colorectal, head and neck, lung, and myeloma cancer types or abnormal results suspicious for cancer in these disease areas. We assessed differences in diagnosis and/or treatment recommendations between the initial and second opinions, characterizing types, sources, and expected clinical impacts of observed differences. Our methods are reported according to the STROBE checklist (Appendix A). Ethics Statement: This study was reviewed by the Institutional Review Board of Memorial Sloan Kettering Cancer Center and obtained a waiver of informed consent.

### 2.2 Study setting and data sources

We used medical and administrative records from Memorial Sloan Kettering Cancer Center (MSK). These included records and notes from patients' outside providers.

MSK is a high‐volume cancer center with over 700,000 patient visits annually. MSK treats 400+ sub‐types of cancer. For patients seeking a second opinion, the process involves collection and review of outside records, repeat or additional diagnostic tests as needed, and clinical consultation with subspecialty physicians (surgeon, medical oncologist, and/or radiation oncologist). Complex cases are presented at a multidisciplinary tumor board for additional expert input.

### 2.3 Study sample

We evaluated 120 cases, 30 each from colorectal, head and neck, and lung cancers, and multiple myeloma. These 4 disease types represent common (colorectal, lung) and rarer (head and neck, myeloma) malignancies. We chose 30 cases per disease a‐priori as the approximate threshold for reaching data saturation (e.g., various subtypes of each disease, the range and characteristics of diagnostic and treatment recommendations from outside and MSK providers); after reviewing 30 cases, we confirmed with the reviewing physicians that the cases they had seen were broadly representative of the kinds of second opinions that usually present.

We defined second opinion consultations as those where patients had completed a “first opinion” (i.e., received a diagnosis and had a well‐documented treatment plan from an outside treating clinician). We defined an outside treating clinician as a non‐MSK medical oncologist, radiation oncologist, or surgeon qualified to provide a treatment recommendation; for colorectal cancer, gastroenterologists were also considered treating clinicians, given their common involvement in the treatment recommendations for this disease. We limited the analysis to newly diagnosed cases of cancers or abnormal results suspicious for cancer; cases of recurrent disease were excluded. We excluded cases where treatment had already been initiated (e.g., patient had surgery but radiation pending); for myeloma however, we included cases where at most one cycle of systemic treatment had been given because the vast majority of patients presenting for a second opinion had recently initiated treatment in this way, and the treatment plan still allowed for modifications based on second opinion recommendations. For each disease type, specific diagnoses seen by the same specialists were excluded: skin cancers for head & neck, anything except colon and rectal cancer for colorectal (e.g., pancreatic, liver, gastric cancers), anything except lung cancers for lung (e.g., mediastinal tumors), and anything except myeloma and its precursors for myeloma.

We used a sequential approach to patient selection, starting on January 1, 2018 through June 2018. We chose these dates to ensure adequate time for the second opinion, including new diagnostic tests and other clinical consultations, to be fully rendered. Every new patient presenting on or after this date to any of the subspeciality physician members of a disease management team (DMT: colorectal, head and neck, lung, and myeloma) was reviewed for possible inclusion; as cases were excluded we moved forward in time, until we found 30 cases in each disease‐type meeting inclusion criteria. We included patients who ultimately underwent treatment at MSK and those who sought treatment elsewhere. We excluded patients for whom the study reviewer was the primary physician for the second opinion and patients who were employees of MSK. (See Appendix B Consort Diagram).

### 2.4 Data abstraction

For each disease type, two attending physicians with subspecialty training and clinical experience reviewed 30 cases. Each pair included a medical and surgical oncologist, except for myeloma where both reviewers were medical oncologists. The reviewers were chosen by convenience; other clinical specialties were not purposefully excluded. All case reviews occurred between August 2020 and March 2021.

A comprehensive data collection guide was developed by the study team prior to case reviews. This guide was informed by the literature^3^, ^8^, ^10^, ^12^ and aimed to capture a large number of potentially relevant diagnostic and treatment details. Each physician pair then reviewed their first 5–10 cases with members of the study team present. Through discussion of these early cases, the guide was iteratively refined and finalized (Appendix C). When changes to the data collection guide were made, reviewers re‐assessed earlier cases to ensure fidelity. At the end of data collection, the study team re‐assessed the full dataset for potential coding inconsistencies; all reviewer pairs then regrouped to ensure coding consistency. Additional data on patients' demographic characteristics and other details of the patients' cases were obtained from MSK electronic records.

For each case, the reviewers independently examined the medical records to complete two questions indicating whether the second opinion involved changes to the diagnosis or treatment plan. Responses to these 2 questions were used to assess inter‐rater reliability, with differences resolved through discussion. All other questions were answered jointly.

### 2.5 Key variables on clinical impact

We iteratively developed consensus definitions and examples of “clinically meaningful” diagnostic and treatment changes through discussion among reviewers during the first 5–10 case reviews. Specifically, in our original Data Collection Guide, we had a 4‐level response of No Change, Minor, Moderate, Major. However, through the first 5–10 questions, this was revised to 2‐level, to try to better reflect a binary judgment about whether there was a clinically meaningful impact on prognosis or morbidity. Furthermore, we refined the language of the definitions for what counted as a clinically meaningful change, and the associated examples. The purpose was to standardize the definition across the four diseases, and to attempt to mitigate the risk of institutional bias among our reviewers toward overcalling clinically meaningful changes. The final definitions are as follows:

- Clinically meaningful changes in diagnosis are those leading to clinically meaningful changes in treatment. For example, a clinically meaningful diagnostic change might be review of radiology images which changed the clinical stage, leading to a change in treatment that was expected to impact outcomes. By contrast, a change in diagnosis that was NOT clinically meaningful would be a change in interpretation of radiology images that did NOT lead to a change in treatment, even if the new interpretation meant that the prognosis was worse (e.g., the tumor was more invasive, or there were more lymph nodes identified).
- Clinically meaningful changes in treatment are those with expected impacts on the outcomes of prognosis, short‐term morbidity, and/or long‐term morbidity. For example, a clinically meaningful change in treatment would be a change in the sequence of chemoradiation and surgery such that the expected short‐term morbidity or prognosis was improved. Conversely, a change in treatment that was NOT clinically meaningful would be a change in chemotherapy regimen with NO expected impact on morbidity or prognosis.

Notably, in assessing impacts on morbidity and prognosis, reviewers estimated expected impact rather than actual impact, since collecting actual outcomes of care was beyond the scope of this study, and, in most cases, would require an extended follow‐up period. Accordingly, even when patients stayed at MSK for treatment, reviewers were instructed not to look past early 2018 for any outcomes of care for these patients, but were instead asked to imagine a generic patient in the given scenario and make a judgment about the expected clinical impact. This approach offers the advantage of generalized, predicted outcomes rather than actual outcomes of specific patients, who may respond heterogeneously to treatment.

### 2.6 Analysis

To assess inter‐rater agreement, we calculated Cohen's Kappa scores for whether the reviewers identified (1) a change in diagnosis and (2) a clinically meaningful change in treatment (binary outcomes).

We performed descriptive analyses of items in the data collection guide overall and by cancer type. For cases with clinically meaningful changes in diagnosis or treatment, subsequent analyses quantified why the diagnosis changed and the reasons why these cases were considered clinically meaningful and associated clinical details, such as modality changes, changes between palliative and curative intent, and guideline concordance of the first opinion.

## 3 RESULTS

### 3.1 Study sample

Appendix B shows the number of patients screened and reasons for exclusion; lack of a well‐documented first opinion treatment plan was the most common reason for exclusion. There were 120 patients in our final analytic sample, 30 each in colorectal, head and neck, lung, and multiple myeloma. The mean (median) age ranged from 54 (57) years for head and neck patients to 64 (67.5) years for lung patients. Overall, 42% of the patients in our sample were female, 74% were white, and 3% were Hispanic or Latino. Fifty‐six percent were privately insured, 8% spoke a primary language other than English, and 63% were married or had a life/domestic partner (Table 1).

**TABLE 1:** Patient characteristics.

	Colorectal cancer (*n* = 30)	Head and neck cancer (*n* = 30)	Lung cancer (*n* = 30)	Myeloma (*n* = 30)	Total (*n* = 120)
Mean (median) age at visit (years)	61 (61.5)	54 (57)	65 (67.5)	60 (61.5)	60 (60.5)
Female	9 (30%)	12 (40%)	12 (40%)	17 (57%)	50 (42%)
Race					
Asian‐Far East/Indian	5 (17%)	4 (13%)	4 (13%)	1 (3%)	14 (12%)
Black or African American	2 (7%)	0 (0%)	0 (0%)	4 (13%)	6 (5%)
No value entered/Pt refused to answer	0 (0%)	3 (7%)	2 (7%)	1 (3%)	6 (5%)
White	22 (73%)	21 (80%)	24 (80%)	23 (77%)	90 (75%)
Other	1 (3%)	2 (0%)	0 (0%)	1 (3%)	4 (3%)
Ethnicity: Hispanic or Latino	0 (0%)	0 (0%)	0 (0%)	3 (10%)	3 (3%)
Payer type					
Medicaid	1 (3%)	2 (7%)	2 (7%)	3 (10%)	8 (7%)
Medicare	12 (40%)	6 (20%)	16 (53%)	10 (33%)	44 (37%)
Private	17 (57%)	22 (73%)	11 (37%)	17 (57%)	67 (56%)
Self‐pay	0 (0%)	0 (0%)	1 (3%)	0 (0%)	1 (1%)
Primary language other than English	2 (7%)	1 (3%)	3 (10%)	2 (7%)	9 (8%)
Married/life/domestic partner	18 (60%)	17 (57%)	18 (60%)	20 (67%)	74 (62%)
Outside provider with NCI designation	6 (20%)	8 (27%)	5 (17%)	8 (27%)	27 (23%)

### 3.2 Treatment changes

**FIGURE 1:**
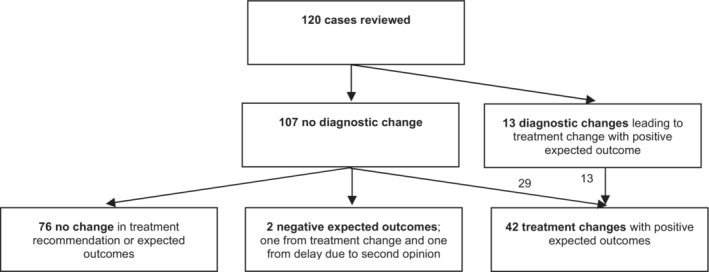
Summary of all diagnostic and treatment changes. See Table 5 for details of diagnostic changes. See Tables 2 and 3 for details of treatment changes.

There was near‐perfect agreement between the reviewers' independent, pre‐consensus assessments of clinically meaningful changes in treatment (Cohen's Kappa: 0.945, 95% confidence interval: 0.885–1). Of 120 cases, 2 had *negative* expected impacts from the change in treatment, and 42 had *positive* expected impacts (Figure 1, Table 2, Appendix D).

**TABLE 2:** Proportion of expected impactful changes in treatment recommendations on outcomes of morbidity and prognosis.

	Colorectal cancer (*n* = 30)	Head and neck cancer (*n* = 30)	Lung cancer (*n* = 30)	Myeloma (*n* = 30)	Total (*n* = 120)
No expected impact on morbidity or prognosis^a^	21 (70%)	13 (43%)	19 (63%)	23 (77%)	76 (63%)
Expected positive impact on morbidity and/or prognosis^b^	7 (23%)	17 (57%)	11 (37%)	7 (23%)	42 (35%)
Expected negative impact on morbidity^c^	2 (7%)	0 (0%)	0 (0%)	0 (0%)	2 (2%)

^a^ No expected impact = no change in treatment recommendation, OR change in treatment recommendation but without expected positive or negative impact on outcomes.

^b^ Expected positive impact = change in treatment recommendation with expected positive impact on outcomes from change.

^c^ Includes one case of change in treatment recommendation with negative impact, and one case without a change in treatment recommendation, but with negative impact from delay associated with seeking second opinion (see Results for further detail, and Appendix for full case descriptions).

In the 2 cases with *negative* expected impacts, the anticipated result was *worse short‐term morbidity* with *no change in long‐term morbidity or prognosis*. One involved a treatment change to what the reviewers judged was an inferior chemotherapy regimen. The other involved no treatment change per se, but the decision to delay treatment while completing the second opinion led to an episode of bleeding from the patient's tumor, requiring transfusion.

Among the 42 cases with *positive* expected impacts, the rates of treatment change varied by disease type: 7/30 for colorectal, 17/30 for head and neck, 11/30 for lung, and 7/30 for myeloma, 23%–57% across 4 disease types (Table 2). These 42 include 13 cases with diagnostic changes, plus 29 additional cases where MSK confirmed the initial diagnosis but altered the treatment plan in ways expected to *improve prognosis and/or morbidity*.

### 3.3 Expected outcomes among 42 positive expected impact cases

All 42 cases had positive expected impacts on short‐ and/or long‐term morbidity. In addition, 11 had expected improvements in prognosis. None involved worse long‐term morbidity or worse prognosis. However, 5 involved worse short‐term morbidity: One head and neck patient had a tradeoff of worse short‐term morbidity in favor of better long‐term morbidity, and 4 lung patients had a tradeoff of worse short‐term morbidity in favor of better prognosis. Variations were observed by disease type (Table 3):

- Improved short‐term morbidity was expected in 36 cases (7 colorectal, 16 head and neck, 7 lung, 6 myeloma, 20%–53%).
- Improved long‐term morbidity was expected in 26 cases (5 colorectal, 13 head and neck, 6 lung, 2 myeloma, 7%–43%).
- Improved prognosis was expected in 11 cases (3 head and neck, 7 lung, 1 myeloma, 0%–23%).

**TABLE 3:** Expected impact on outcomes of morbidity and prognosis of treatment recommendation changes, among the 42 patients with positive impacts on outcomes.^a^

	Colorectal cancer (*n* = 30)	Head and neck cancer (*n* = 30)	Lung cancer (*n* = 30)	Myeloma (*n* = 30)	Total (*n* = 120)
Expected impact on prognosis					
Better	0 (0%)	3 (10%)	7 (23%)	1 (3%)	11 (9%)
Worse	0 (0%)	0 (0%)	0 (0%)	0 (0%)	0 (0%)
Expected impact on short‐term morbidity					
Better	7 (23%)	16 (53%)	7 (23%)	6 (20%)	36 (30%)
Worse^b^	0 (0%)	1 (3%)	4 (13%)	0 (0%)	5 (4%)
Expected impact on long‐term morbidity					
Better	5 (17%)	13 (43%)	6 (20%)	2 (7%)	26 (22%)
Worse	0 (0%)	0 (0%)	0 (0%)	0 (0%)	0 (0%)

^a^ Data are from overlapping cases; all 42 cases with positive expected impact had improvement in short‐term and/or long‐term morbidity (23%–57% across 4 disease types); 11 of these 42 also had positive expected impact on prognosis.

^b^ In the 5 cases with expected worse short‐term morbidity, this was a tradeoff in favor of better long‐term morbidity (1 in head and neck) or better prognosis (4 in lung). Note that this table does not include the 2 cases with negative impact from having sought a second opinion, both of which resulted in worse short‐term morbidity.

### 3.4 Modality‐ and case‐level shifts

Table 4 shows treatment changes by modality. Across the 42 positive‐impact cases, 6 involved adding surgery or increasing its extent, whereas 21 involved eliminating or reducing surgery. Other modality changes were more evenly divided between additions and increases (11 for radiation, 11 for systemic therapy) and eliminations and reductions (9 for radiation, 11 for systemic therapy). Other notable case‐level changes pertain to de‐escalation with improved expected morbidity:

- Nine cases (3 head and neck, 6 myeloma) involved a shift from treatment to no treatment with recommended observation of abnormal findings.
- Eight cases (3 colorectal, 3 head and neck, 2 lung) had a shift from surgical to non‐surgical management.
- Two cases (1 head and neck, 1 myeloma) involved a shift from systemic drug treatment to local treatment with surgery and/or radiation.

**TABLE 4:** Shifts in treatment modality^a^ from second opinion among the 42 patients with positive impacts on outcomes.

	Colorectal cancer (*n* = 30)	Head and neck cancer (*n* = 30)	Lung cancer (*n* = 30)	Myeloma (*n* = 30)	Total (*n* = 120) (% is out of 120 except where noted)
Surgery					
Add	0 (0%)	3 (10%)	1 (3%)	X	4 (4%, *n* = 90)
Increase	1 (3%)	1 (3%)	0 (0%)	X	2 (2%, *n* = 90)
Subtract^b^	3 (10%)	6 (20%)^b^	2 (7%)	X	11 (12%, *n* = 90)
Decrease	2 (7%)	6 (20%)	2 (7%)	X	10 (11%, *n* = 90)
Radiation					
Add	2 (7%)	2 (7%)	4 (13%)	1 (3%)	9 (8%)
Increase	0 (0%)	2 (7%)	0 (0%)	0 (0%)	2 (2%)
Subtract	1 (3%)	1 (3%)	3 (10%)	0 (0%)	5 (4%)
Decrease	0 (0%)	4 (13%)	0 (0%)	0 (0%)	4 (3%)
Systemic therapy					
Add	3 (10%)	4 (13%)	0 (0%)	0 (0%)	7 (6%)
Increase	0 (0%)	0 (0%)	4 (13%)	0 (0%)	4 (3%)
Subtract^b^	0 (0%)	1 (3%)	0 (0%)	7 (23%)^b^	8 (7%)
Decrease	1 (3%)	2 (7%)	0 (0%)	0 (0%)	3 (3%)

^a^ Definitions are as follows: Adding/ subtracting a modality (surgery, radiation, or systemic therapy) means going from not using the modality in the 1st opinion to using it in the second (adding), or taking it away (subtracting). Increasing/ decreasing means going from some amount in the 1st opinion to more in the second (increase), or less in the second (decrease).

^b^ In 3/6 head & neck cases surgery was subtracted and no treatment recommended; In 6/7 myeloma cases systemic treatment was subtracted and no treatment recommended (9 total cases changed to no treatment/observation recommended).

In 5 cases (1 head and neck, 3 lung, 1 myeloma) the outside treatment recommendation involved non‐guideline concordant care judged to be unhelpful or potentially harmful; in these cases, MSK recommended a shift to guideline‐concordant care. Other treatment changes involved clinical trials at MSK (3 head and neck, 1 lung), shifts from curative to palliative treatment (1 colorectal, 1 head and neck), or shifts from palliative to curative (2 lung).

### 3.5 Changes in diagnosis

There was near‐perfect agreement between the reviewers' independent, pre‐consensus assessments of changes in diagnosis (Cohen's Kappa: 0.952, 95% confidence interval: 0.858–1). Of 120 cases, 13 involved clinically meaningful diagnostic changes leading to treatment changes expected to impact morbidity and/or prognosis (Figure 1). Rates varied by disease: 1/30 cases (3%) for colorectal, 5/30 cases (17%) for head and neck, 3/30 cases (10%) for lung, and 4/30 cases (13%) for myeloma (Table 5, Appendix D).

**TABLE 5:** Frequency, types, and sources of clinically meaningful diagnostic changes recommended after second opinion consultation.^a^

	Colorectal cancer (*n* = 30)	Head and neck cancer (*n* = 30)	Lung cancer (*n* = 30)	Myeloma (*n* = 30)	Total (*n* = 120) (% is out of 120 except where noted)
Number of clinically meaningful diagnostic changes	1 (3%)	5 (17%)	3 (10%)	4 (13%)	13 (11%)
Types of changes to the diagnosis					
More advanced disease	1 (3%)	3 (10%)	0 (0%)	0 (0%)	4 (3%)
Less advanced disease	0 (0%)	1 (3%)	0 (0%)	4 (13%)	5 (4%)
Refinement of diagnosis (not more or less advanced)	0 (0%)	1 (3%)	3 (10%)	0 (0%)	4 (3%)
Information sources for diagnostic changes					
Review of outside pathology	0 (0%)	0 (0%)	0 (0%)	X	0 (0%)
Review of outside radiology	1 (3%)	4 (13%)	0 (0%)	X	5 (6%, *n* = 90)
Review of outside labs	0 (0%)	0 (0%)	0 (0%)	X	0 (0%)
Integrated review of all three diagnostic modalities (pathology, radiology, and labs—Myeloma only)	X	X	X	4 (13%)	4 (13%, *n* = 30)
MSK radiology performed	0 (0%)	0 (0%)	0 (0%)	0 (0%)	0 (0%)
MSK pathology performed (new biopsy)	0 (0%)	0 (0%)	3 (10%)	0 (0%)	3 (3%)
MSK physical exam	0 (0%)	1 (3%)	0 (0%)	0 (0%)	1 (1%)
Advanced diagnostic testing at MSK, including personalized or unique molecular or other testing	0 (0%)	0 (0%)	1 (3%)	0 (0%)	1 (1%)

*Note*: A single case can appear in multiple rows.

Abbreviation: MSK, Memorial Sloan Kettering Cancer Center.

^a^ We defined “clinically meaningful” as those that led to changes in treatment that were expected to be impactful in a positive direction.

Of the 13 cases with clinically meaningful diagnostic changes, 4 involved identification of *more advanced* disease; 5 identified *less advanced* disease; and 4 involved other *diagnostic refinements* (Table 5). Diagnostic changes were most commonly due to review of *outside radiology images* (*n* = 5), *integrated review* of all diagnostic modalities (*n* = 4), and *new MSK pathology (new biopsy*) (*n* = 3). In one case, a *physical exam* at MSK prompted the diagnostic change, and, in another, an *advanced molecular diagnostic test* informed the change.

## 4 DISCUSSION

This study sought to assess the clinical value of second opinion consultations at a high‐volume, tertiary‐care cancer center. Among 120 cases reviewed, the specialty second opinion was rarely inferior to the first, and often offered meaningful diagnostic and treatment changes with better expected patient outcomes (*n* = 42). Most frequently, however, MSK confirmed the outside diagnosis and treatment plan (*n* = 76). This finding is significant, as reassurance is highly valued by patients and is one of the most common reasons for obtaining second opinions.^6^, ^13^, ^14^, ^15^

We also found that only a subset of second opinions leading to meaningful treatment changes (*n* = 42) were due to meaningful diagnostic changes (*n* = 13). While many definitions of second opinion diagnostic change exist,^16^, ^17^, ^18^, ^19^, ^20^, ^21^, ^22^, ^23^, ^24^, ^25^ our findings are in the range of those from studies with similarly conservative definitions of “meaningful” change in both diagnosis and treatment.^3^, ^8^, ^9^ The greater relative frequency of treatment change might be explained by the relative complexity and range of treatment options compared to the complexity of diagnostic evaluation. This information is important in setting expectations for patients, clinicians, and payers.

Among the 42 cases with positive, clinically meaningful treatment changes, all had expected improvement in short and/or long‐term morbidity, and 11 also had improved expected prognosis. This finding suggests that the value of high‐quality second opinions in oncology derives frequently from de‐escalating or modifying treatment in ways that reduce treatment‐associated morbidity, more often than improving prognosis.

Notably, second‐opinion treatment changes generally enhanced or optimized the prior recommendation; initial treatment recommendations rarely ran counter to available guidelines: Only 5 cases had a first opinion that was not guideline concordant and potentially harmful. In all other cases where the reviewers agreed that there were positive treatment changes, the second opinion leveraged subspecialty expertise to offer nuanced changes with expected better outcomes.

Our findings raise several avenues of inquiry. First, although out results suggest subgroup heterogeneity in terms of the clinical value of second opinions, further work should identify which subgroups might benefit most. Second, the value of second opinions may differ for patients with recurrent disease or those already undergoing treatment; these patients constitute a large portion of second opinions at high‐volume specialty centers but were not included in this study. Third, the extent to which specialty second opinion recommendations are portable to community settings is unknown. Most cancers are diagnosed and treated in community settings, and the ability to transport recommendations from a specialty cancer center to the community may improve care for these patients. Fourth, equitable access to second opinion consultations is a critical issue. As cancer centers expand their telemedicine and remote second opinion programs, the value and equity of these programs must be continuously assessed.^3^, ^26^, ^27^

There are limitations to consider in interpreting our results. First, there is a risk for institutional confirmation bias, since our methods required physician reviewers to make somewhat subjective judgments about changes and expected outcomes among cases from their own institution. We note for example, there were 2 cases where decisions about outcomes hinged on clinical trial participation; these may be subject to particular difficult attribution. However, to counter this potential bias, we used an a‐priori conservative approach for all the cases to identify only clinically meaningful changes expected to impact outcomes. We also followed established methods in quality assessment research,^28^, ^29^, ^30^ developed our data collection guide iteratively with reviewer input, and used two expert physician reviewers from different specialties for each disease type, who were shown to have strong agreement. We do note that high Kappa scores could also be a reflection of institutional homogeneous practices. Second, the generalizability of the findings may be limited as we examined second opinions at a single specialty cancer center: practice patterns and patient populations may differ elsewhere. Third, the ranges of estimates in clinically meaningful changes should be viewed as exploratory rather than definitive, since we only examined 30 cases from each of 4 disease types. Fourth, due to the small numbers of patients in each of the four diseases, comparisons of the frequency of changes in management across rare and common diseases or other subgroups such as treatment type could not legitimately be performed. Fifth, our review was retrospective using electronic medical record data. Inherent in all such studies are limitations including the possibility of incomplete documentation of information from first‐opinion providers.

This study provides an in‐depth analysis of the clinical value of second opinion consultations in oncology at a high‐volume cancer center. As subspecialty second opinion consultations may be resource‐intensive, it is critical to determine which sub‐groups would most benefit from full second opinion consultations and when more narrowly‐focused diagnostic or treatment reviews are sufficient. Equally important, we must develop methods to broaden access to specialty expertise, bringing the referral center and community closer together to support front‐line clinicians and their patients.

## AUTHOR CONTRIBUTIONS

**Allison Lipitz‐Snyderman:** Conceptualization, data curation, formal analysis, investigation, methodology, project administration, resources, validation, visualization, writing ‐ original draft, and writing ‐ review and editing. **Susan Chimonas:** Conceptualization, data curation, formal analysis, investigation, methodology, project administration, resources, validation, visualization, writing ‐ original draft, and writing ‐ review and editing. **Sham Mailankody:** Conceptualization, data curation, methodology, and writing ‐ review and editing. **Michelle Kim:** Conceptualization, data curation, project administration, resources, software, validation, visualization, and writing ‐ review and editing. **Nicholas Silva:** Data curation, project administration, resources, software, visualization, and writing ‐ review and editing. **Anuja Kriplani:** Data curation, methodology, and writing ‐ review and editing. **Leonard B. Saltz:** Data curation, methodology, and writing ‐ review and editing. **Smita Sihag:** Data curation, methodology, and writing ‐ review and editing. **Carlyn Rose Tan:** Data curation, methodology, and writing ‐ review and editing. **Maria Widmar:** Data curation, methodology, and writing ‐ review and editing. **Marjorie Zauderer:** Data curation, methodology, and writing ‐ review and editing. **Saul Weingart:** Conceptualization, methodology, supervision, and writing ‐ review and editing. **Wendy Perchick:** Conceptualization, methodology, resources, supervision, and writing ‐ review and editing. **Benjamin R. Roman:** Conceptualization, data curation, formal analysis, investigation, methodology, project administration, resources, validation, visualization, writing ‐ original draft, and writing ‐ review and editing.

## CONFLICT OF INTEREST STATEMENT

The authors acknowledge the potential for confirmation bias to influence findings from review of cases from their own institution, although a conservative approach to expected outcome assessment was taken. The authors report the following disclosures: In the last 3 years, Dr. Zauderer has received consulting fees from Takeda, GlaxoSmithKline, Aldeyra Therapeutics, Novocure, and Atara and honoraria from Research to Practice, Medical Learning Institute, OncLive and Expert Connect. Memorial Sloan Kettering receives research funding from the Department of Defense, the National Institutes of Health, Precog, GlaxoSmithKline, Epizyme, Polaris, Sellas Life Sciences, Bristol Myers Squibb, Millenium/Takeda, Curis, and Atara for research conducted by Dr. Zauderer. Dr. Zauderer serves as Chair of the Board of Directors of the Mesothelioma Applied Research Foundation, uncompensated. No other authors have disclosures to report.

## FUNDING INFORMATION

This study was supported in part by the Memorial Sloan Kettering Cancer Center Support Grant P30 CA008748 from the National Institutes of Health/National Cancer Institute.

## ETHICS STATEMENT

This study was reviewed by the Institutional Review Board of Memorial Sloan Kettering Cancer Center and obtained a waiver of informed consent.

## Supporting information


Appendix A.

Appendix B.

Appendix C.

Appendix D.

## Data Availability

Data sharing is not applicable to this article as no new data were created or analyzed in this study.
