# Supplementary material for: Clinical value of second opinions in oncology: A retrospective review of changes in diagnosis and treatment recommendations
Source: Cancer Med. 2023 Feb 3;12(7):8063–72. doi: 10.1002/cam4.5598 (PMC10134380; doi:10.1002/cam4.5598)
Supplement: Supplementary file 2 — Appendix B. [file CAM4-12-8063-s002.docx]

**Appendix B. Consort Diagram for Study Sample**

Assessed for eligibility (n = 1658)

Previous MSK patient seen in the past for a different cancer (n = 78)

- Colorectal, n = 16
- Head and neck, n = 17
- Lung, n = 45
- Myeloma, n = 0

120 cases included in the review

N = 1033

316

N = 991

304274

Received cancer treatment prior to second opinion consultation at MSK (n = 319)

- Colorectal, n = 131
- Head and neck, n = 27
- Lung, n = 118
- Myeloma, n = 43

No documented treatment plan from prior treating clinician (n = 552)

- Colorectal, n = 140
- Head and neck, n = 102
- Lung, n = 232
- Myeloma, n = 78

N = 439

Cancer recurrence (n = 42)

- Colorectal, n = 16
- Head and neck, n = 11
- Lung, n = 9
- Myeloma, n = 6

Presented for opinion regarding an excluded tumor type; see methods (n= 387)

- Colorectal, n = 325
- Head and neck, n = 36
- Lung, n = 25
- Myeloma, n = 1

MSK employee or reviewer was involved in the patient’s care (n = 63)

- Colorectal, n = 21
- Head and neck, n = 8
- Lung, n = 2
- Myeloma, n = 32

No documented diagnosis from prior treating clinician (n = 97)

- Colorectal, n = 24
- Head and neck, n = 37
- Lung, n = 20
- Myeloma, n = 16

N = 1130

N = 1193

N = 1580
