# Supplementary material for: Clinical value of second opinions in oncology: A retrospective review of changes in diagnosis and treatment recommendations
Source: Cancer Med. 2023 Feb 3;12(7):8063–72. doi: 10.1002/cam4.5598 (PMC10134380; doi:10.1002/cam4.5598)
Supplement: Supplementary file 3 — Appendix C. [file CAM4-12-8063-s001.docx]

**Appendix C: Data Collection Guide**

Prior to data collection, the two reviewers were asked to independently read the patient’s Electronic Medical Record for several minutes. They can voice their findings to each other (“take a look at the assessment and plan from the note on January 10th”) but not yet their interpretation of these findings for diagnosis or treatment.

1. Answer independently to calculate Kappa - Was there a change in diagnosis?

- No change (confirm outside diagnosis) - The second opinion diagnosis affirms the accuracy of the first opinion’s diagnosis/diagnostic plan, or recommends only trivial modifications.

Examples:

- The diagnosis and staging of the patient’s cancer are correct
- Review of outside pathology describes a new cell feature or tumor marker that does not change the diagnosis
- Review of outside radiology describes the cancer with more detail, but without changing the diagnosis
- Change - The second opinion changes the first diagnosis through a clarification, refinement, or addition of information. Note that the change MAY or MAY NOT impact the treatment recommendation- this will be assessed later.

Examples:

- Change in tumor pathologic grade, feature, cell type, etc, based on review of outside pathology or new biopsy material
- Change in tumor stage or refinement of the extent of disease, based on review of previous radiology or new physical exam/ new diagnostic test.
- Additional diagnostic test recommended that was not recommended from the first opinion, which rules out metastases, clarifies the extent of disease, or adds molecular diagnostic information

2) Jointly discuss question 1 to arrive at consensus:

- Change in diagnosis ⮚ CONTINUE TO #3
- No change in diagnosis ⮚ SKIP TO #6

3) Jointly discuss - Which category of diagnostic clarification or change? [check all that apply]

1. Change in clinical/ radiologic TNM Staging

- T stage
- N stage

1. Change in Pathologic TNM Staging

- T stage
- N stage

c. Change in pathologic features

- - - Different cell type
    - Different grade or categorization
    - Different morphological features
    - MSK performed or looked at features NOT DONE OUTSIDE

1. Other: Please specify [free text]

4) Jointly discuss - Did the MSK diagnosis identify:

- More advanced disease
- Less advanced disease
- Other diagnostic refinement (not more or less advanced disease)

5) Jointly discuss - Was the change in diagnosis due to [check all that apply]:

- MSK review of history
- MSK review of outside radiology
- MSK review of outside pathology
- MSK review of labs
- MSK integrated review of all diagnostic modalities (pathology, radiology, and labs)
- New MSK physical exam
- New MSK pathology
- New MSK radiology
- New MSK laboratory
- Other: Please specify [free text]

6) Jointly discuss - Was a unique or advanced diagnostic test performed at or ordered by MSK, including pathology stains/slides or molecular tests, or MSK-IMPACT/ FoundationOne testing?

- Yes: Please specify which, and its potential significance [free text]
- No

7) Answer independently to calculate Kappa - Was there a change in treatment recommendation?

- No change in treatment recommendation - The second opinion diagnosis confirms the appropriateness of the first opinion’s therapeutic plan or recommends only trivial modifications with no expected impact on outcomes of short-term morbidity, long-term morbidity, and/or prognosis.

Examples:

- The current chemoradiation regimen is the appropriate first-line treatment.
- The current chemoradiation regimen is appropriate, but recommend minor change in drug, dosing, frequency, etc that is not expected to impact outcomes.
- The first opinion suggested observation for an abnormal mass that is possibly cancerous, but mentioned surgery as an option; the second opinion agrees with observation.
- Change - The second opinion recommends altering the treatment plan, through an adjustment of details within the same treatment modality, addition/subtraction of modality or change in the sequence of treatment modalities, or clarification of a choice between different treatment options. Note that to be counted as a change, there MUST BE an expected impact on outcomes of short-term morbidity, long-term morbidity, and/or prognosis.

Examples:

- The current regimen of chemotherapy is reasonable but would recommend administering drug weekly, as this has a more favorable toxicity profile.
- The first opinion suggested a curative treatment regimen, but mentioned palliative treatment as an option; the second opinion helped the patient decide on palliative care given the extent of disease.
- The second opinion recommends a change in the extent of surgery to avoid morbidity, with same expected prognosis.
- The current planned regimen and sequence of surgery, radiation, and chemotherapy is not appropriate or guideline concordant; the second opinion recommends a guideline concordant plan.
- Although first opinion plan for surgery followed by possible adjuvant treatments was guideline concordant, chemoradiation recommended instead of surgery due to lower morbidity

8) Jointly discuss question 7 to arrive at **consensus:**

- No change in treatment recommendation ⮚ SKIP TO #15
- Change in treatment recommendation ⮚ CONTINUE TO #9

9) Jointly discuss – If there was also a change in diagnosis above, was the change in treatment due to the change in diagnosis?

- Yes
- No

10) Jointly discuss - What is the expected clinical impact of the change in treatment? Please imagine a cohort of similar patients in this patient’s scenario, and make a judgment about the expected clinical impact on that cohort, on average.

a. Change in **prognosis**

- - - better
    - worse
    - same
    - unknown

b. Change in **short-term treatment related morbidity or toxicity**:

- - - better
    - worse
    - same
    - unknown

c. Change in **long-term morbidity**:

- - - better
    - worse
    - same
    - unknown

11) Jointly discuss - What modality changes were made, if any?

NOTES:

Decrease = “change in *amount* of surgery/radiation, *amount* of same drug, or change in *regimen* (drug) such that it represents a *de-escalation* with potentially less morbidity”

Increase = “change in *amount* of surgery or radiation, or *amount* of same drug, or change in *regimen* (drug) such that it represents an *escalation* with potentially worse morbidity (usually with the trade-off of improved prognosis)”

a. Surgery:

- No change, or change that did not reflect “amount”
- Add
- Increase
- Subtract
- Decrease

b. Radiation:

- No change, or change that did not reflect “amount”
- Add
- Increase
- Subtract
- Decrease

c. Systemic therapy:

- No change, or change that did not reflect “amount”
- Add
- Increase
- Subtract
- Decrease

12) Jointly discuss - Was the first opinion treatment recommendation non-guideline concordant care judged to be unhelpful or potentially harmful?

- Yes
- No

13) Is the change in treatment a shift from:

- Palliative to curative treatment
- Curative to palliative treatment
- Neither

14) Is the change in treatment due to enrollment in a clinical trial at MSK?

- Yes
- No

15) Jointly discuss - Is there any *negative* expected impact from the second opinion process, either because of negative impact of a change in treatment recommendation from the second opinion, or due to the process of obtaining the opinion?

- Yes ⮚ CONTINUE TO #16
- No ⮚ SKIP TO #18

16) Jointly discuss - Is the negative expected impact due to [select all that apply]:

- MSK change in treatment recommendation ⮚ SKIP TO #18 [since the expected change in clinical outcomes would be captured above in #10]
- Other: Please specify (e.g. delay in treatment) [free text] ⮚ CONTINUE TO #17

17) Jointly discuss - What is the expected change in clinical outcomes from the negative event or factor identified in #16?

a. Change in **prognosis**

- - - better
    - worse
    - same
    - unknown

b. Change in **short-term treatment related morbidity or toxicity**:

- - - better
    - worse
    - same
    - unknown

c. Change in **long-term morbidity**:

- - - better
    - worse
    - same
    - unknown

18) Jointly discuss - Could the patient’s initial second opinion consultation have been conducted via telemedicine?

- All ⮚ SKIP TO #20
- Some ⮚ CONTINUE TO #19
- None ⮚ CONTINUE TO #19

19) Jointly discuss - Why was an in-person visit essential? Select all that apply:

- A physical exam was needed
- A test needed to be conducted onsite
- Other: Please specify [free text]

20) Jointly formulate a CASE DESCRIPTION/NARRATIVE SUMMARY of the case, with [free text]:

- Initial diagnosis (pre-second opinion)
- Initial treatment plan (pre-second opinion)
- MSK diagnosis (if different, otherwise can say no change)
- MSK treatment plan (if different, otherwise can say no change)
- If change in treatment with expected change in outcomes of prognosis, short-term or long-term morbidity, explain reasoning for the expected change in each affected outcome
