# Supplementary material for: Clinical value of second opinions in oncology: A retrospective review of changes in diagnosis and treatment recommendations
Source: Cancer Med. 2023 Feb 3;12(7):8063–72. doi: 10.1002/cam4.5598 (PMC10134380; doi:10.1002/cam4.5598)
Supplement: Supplementary file 4 — Appendix D. [file CAM4-12-8063-s004.docx]

**Appendix D**

**Cases with Clinically Meaningful Changes from MSK Second Opinions**

**2 cases with negative expected impacts on outcome**

**Colorectal Case 1**

Short-term morbidity: worse

Long-term morbidity: no change/unknown

Prognosis: no change/unknown

A 59 year old man came to MSK with an outside diagnosis of metastatic colorectal cancer, Stage IV, with multiple liver metastases as well as extrahepatic disease. The outside recommendation was for chemotherapy following NCCN (National Comprehensive Cancer Network) guidelines. MSK did not meaningfully change the diagnosis. However, MSK changed the treatment recommendation to chemotherapy with a nonstandard regimen. The MSK change in treatment recommendation was expected to negatively impact short-term morbidity due to increased toxicity. The MSK change in treatment recommendation was not expected to impact prognosis or long-term morbidity.

**Colorectal Case 2**

Short-term morbidity: worse

Long-term morbidity: no change/unknown

Prognosis: no change/unknown

A 91 year old man came to MSK with an outside diagnosis of colon cancer. The outside recommendation was for surgery, and he was medically cleared for the procedure. MSK did not meaningfully change the diagnosis. However, given patient frailty, comorbidities, and age, MSK conducted additional pre-operative evaluation and considered non-surgical options, which delayed starting treatment. During the delay, the patient had an episode of bleeding requiring a transfusion. MSK ultimately confirmed the patient’s need for surgery, so the MSK second opinion did not change the treatment recommendation. The delay at MSK was expected to negatively impact short-term morbidity due to the bleeding episode but was not expected to impact prognosis or long-term morbidity.

**42 cases with positive expected impacts on outcome**

**Colorectal Case 1**

Short-term morbidity: better

Long-term morbidity: better

Prognosis: no change/unknown

A 67 year old man came to MSK with an outside diagnosis of rectal cancer, Stage I. The outside recommendation was for surgery. MSK did not meaningfully change the diagnosis, but did change the treatment recommendation to chemoradiation, due to patient preference to avoid surgery. The MSK change in treatment recommendation was expected to positively impact short- and long-term morbidity since surgery could be avoided. The MSK change in treatment recommendation was not expected to impact prognosis.

**Colorectal Case 2**

Short-term morbidity: better

Long-term morbidity: no change/unknown

Prognosis: no change/unknown

A 46 year old man came to MSK with an outside diagnosis of nearly obstructing colon cancer, Stage IV. The outside recommendation was for standard chemotherapy with bevacizumab. MSK did not meaningfully change the diagnosis. However, MSK changed the treatment recommendation to standard chemotherapy without bevacizumab, given the possible need for surgery due to possible obstruction and the bevacizumab’s side effects on wound healing after surgery. The MSK change in treatment recommendation was expected to positively impact short-term morbidity due to reducing possible surgical morbidity. The MSK change in treatment recommendation was not expected to impact prognosis or long-term morbidity.

**Colorectal Case 3**

Short-term morbidity: better

Long-term morbidity: better

Prognosis: no change/unknown

A 37 year old man came to MSK with an outside diagnosis of rectal cancer, Stage III. The outside recommendation was for chemoradiation followed by surgery. MSK did not meaningfully change the diagnosis. However, MSK changed the treatment recommendation to total neoadjuvant therapy (radiation and chemotherapy) with the goal of non-operative management. The MSK change in treatment recommendation was expected to positively impact short- and long-term morbidity due to potentially avoiding surgery. The MSK change in treatment recommendation was not expected to impact in prognosis.

**Colorectal Case 4**

***Change in diagnosis**

Short-term morbidity: better

Long-term morbidity: better

Prognosis: no change/unknown

A 75 year old man came to MSK with an outside diagnosis of rectal cancer, Stage III, and a history significant for prostate cancer treated with radiation and prostatectomy. The outside recommendation was for radiation followed by surgery. MSK changed the diagnosis based upon review of outside radiology, noting higher T stage. Based on the change in diagnosis, MSK changed the treatment recommendation to more extensive surgery after pre-operative treatment. MSK also recommended pre-operative chemotherapy instead of radiation due to previous radiation to the area. The MSK change in treatment recommendation was expected to positively impact short- and long-term morbidity due to decreased toxicity from the pre-operative treatment. It was unknown whether the MSK change in treatment recommendation would be expected to impact prognosis.

**Colorectal Case 5**

Short-term morbidity: better

Long-term morbidity: better

Prognosis: no change/unknown

A 73 year old man came to MSK with an outside diagnosis of colon cancer, Stage 1. The outside recommendation was for surgery with a stoma. MSK did not meaningfully change the diagnosis. However, MSK changed the treatment recommendation to surgery but with the plan to avoid the stoma. The MSK change in treatment recommendation was expected to positively impact short- and long-term morbidity due to avoiding the stoma. The MSK change in treatment recommendation was not expected to impact prognosis.

**Colorectal Case 6**

Short-term morbidity: better

Long-term morbidity: no change/unknown

Prognosis: no change/unknown

A 71 year old woman with very significant comorbidities and frailty (EF 45%, atrial fibrillation, and wheelchair bound), came to MSK with an outside diagnosis of locally advanced T4b colon cancer, with colovesical and colovaginal fistula. The outside recommendation was for surgery, which would require extensive resection of pelvic organs—vagina, bladder, sigmoid, and rectum. MSK did not meaningfully change the diagnosis. However, MSK changed the treatment recommendation to surgical diversion and palliative chemotherapy. It was felt that major resection would not improve survival and would not improve quality of life; in fact major resection would likely result in worse quality of life, given the extent of surgery, and further due to protracted recovery from major operation, she would be unlikely to eventually receive the chemotherapy needed for advanced disease. Conversely, chemotherapy could in fact improve quality of life. The MSK change in treatment recommendation was expected to positively impact short-term morbidity due to avoiding major resection. The MSK change in treatment recommendation was not expected to impact long-term morbidity or prognosis.

**Colorectal Case 7**

Short-term morbidity: better

Long-term morbidity: better

Prognosis: no change/unknown

A 51 year old man came to MSK with an outside diagnosis of early stage rectal cancer. The outside recommendation was for surgery. MSK did not meaningfully change the diagnosis. However, MSK changed the treatment recommendation to total neoadjuvant therapy (radiation and chemotherapy) with the goal of non-operative management. The MSK change in treatment recommendation was expected to positively impact short- and long-term morbidity due to potentially avoiding surgery. The MSK change in treatment recommendation was not expected to impact prognosis.

**Head & Neck Case 1**

***Change in diagnosis**

Short-term morbidity: better

Long-term morbidity: better

Prognosis: better

A 64 year old woman came to MSK with an outside diagnosis of HPV- (head and neck human papillomavirus) associated tonsil SCCA (squamous cell carcinoma), Stage IV. The outside recommendation was for surgery. MSK changed the diagnosis based upon review of outside imaging, noting a retropharyngeal lymph node that upstaged the patient. Based on the change in diagnosis, MSK changed the treatment recommendation to a non-surgical approach, using chemoradiation to treat the retropharyngeal lymph nodes at the same time as the primary tumor. MSK also noted that the outside approach of surgery would have likely been followed by chemoradiation after some delay, once the retropharyngeal lymph nodes were discovered (and considered recurrence). The MSK change in treatment recommendation was expected to positively impact prognosis by providing appropriate, timely treatment of the retropharyngeal nodes. It was also expected to positively impact short- and long-term morbidity by avoiding the outside approach of surgery, followed by retropharyngeal node progression, then likely chemoradiation -- which would have been a larger total treatment package.

**Head & Neck Case 2**

Short-term morbidity: better

Long-term morbidity: better

Prognosis: better

A 66 year old man came to MSK with an outside diagnosis of head and neck HPV-associated tonsil SCCA, Stage IV. The outside recommendation was for neck nodes to be treated with a lower dose of radiation followed by surgery, and for the primary tonsil site to be treated with chemoradiation. MSK did not meaningfully change the diagnosis, but MSK changed the treatment plan because the outside recommendation was not guideline concordant. MSK’s recommendation was for chemoradiation to the primary tonsil site and the neck. The MSK change in treatment recommendation was expected to positively impact prognosis by providing appropriate treatment to the neck; it was also expected to positively impact short-and long-term morbidity by avoiding suboptimal combination of surgery and radiation to the neck.

**Head & Neck Case 3**

***Change in diagnosis**

Short-term morbidity: better

Long-term morbidity: better

Prognosis: no change/unknown

A 56 year old man came to MSK with an outside diagnosis of head and neck HPV-associated base of tongue SCCA, Stage II. The outside recommendation was for primary surgery including bilateral neck dissection. MSK changed the diagnosis upon review of outside imaging, noting extra-nodal extension of lymph nodes. Based on the change in diagnosis, MSK recommendation was for non-surgical treatment with chemoradiation, since primary surgical treatment would have necessitated triple modality therapy (adjuvant chemoradiation) once extra-nodal extension was confirmed on pathology. The MSK change in treatment recommendation was expected to positively impact short- and long-term morbidity by avoiding bilateral neck dissection and triple modality therapy. The MSK change in treatment recommendation was not expected to impact prognosis.

**Head & Neck Case 4**

***Change in diagnosis**

Short-term morbidity: better

Long-term morbidity: no change/unknown

Prognosis: no change/unknown

A 91 year old man came to MSK with an outside diagnosis of head and neck hypopharyngeal SCCA, Stage III. The outside recommendation was for chemoradiation. MSK changed the diagnosis upon review of outside imaging, upstaging by noting pathologic lymph nodes requiring treatment. Upstaging did not change the recommended treatment modality but did change the radiation fields. MSK recommendation was for "Quad Shot” radiation, which despite encompassing a larger field due to the change in diagnosis is a lesser dose with lower morbidity for patients with incurable head and neck cancers. The MSK change in treatment recommendation was expected to positively impact short-term morbidity due to the lower dose of radiation. The MSK change in treatment recommendation was not expected to impact prognosis or long-term morbidity.

**Head & Neck Case 5**

Short-term morbidity: better

Long-term morbidity: no change/unknown

Prognosis: no change/unknown

A 33 year old man came to MSK with an outside diagnosis of head and neck oral tongue SCCA, Stage II. The outside recommendation was for primary surgery with free flap reconstruction. MSK did not meaningfully change the diagnosis, but MSK’s treatment recommendation was for surgery with primary closure instead of a free flap. In this case the free flap was deemed unnecessary to achieve optimal functional outcomes of speech and swallowing. The MSK change in treatment recommendation was expected to positively impact short-term morbidity because of decreased operative time, decreased operative complications and faster recovery. The MSK change in treatment recommendation was not expected to impact prognosis or long term morbidity.

**Head & Neck Case 6**

Short-term morbidity: better

Long-term morbidity: no change/unknown

Prognosis: no change/unknown

A 58 year old man came to MSK with an outside diagnosis of head and neck oral tongue SCCA, Stage I, having had an excisional biopsy of the mass showing no visible residual tumor. The outside recommendation was for additional surgery to the tongue. MSK did not meaningfully change the diagnosis. However, MSK obtained a CT (computerized tomography) scan of the neck to evaluate the lymph nodes. The CT scan was negative, so MSK changed the treatment recommendation to close observation. The MSK change in treatment recommendation was expected to positively impact short-term morbidity by avoiding unnecessary surgery. The MSK change in treatment recommendation was not expected to impact prognosis or long-term morbidity.

**Head & Neck Case 7**

***Change in diagnosis**

Short-term morbidity: better

Long-term morbidity: better

Prognosis: better

A 59 year old man came to MSK with an outside diagnosis of head and neck HPV-associated unknown primary SCCA, Stage I. The outside recommendation was for radiation alone, covering the whole upper aerodigestive tract. MSK changed the diagnosis after performing endoscopic evaluation (not performed outside) and noting a likely tonsil primary tumor. Based on this change in diagnosis, MSK recommendation was for surgical management to the primary tumor and neck nodes, followed by adjuvant radiation therapy. In this case, of initial non-surgical management had been used, MSK noted that radiation alone would have been insufficient. The MSK change in treatment recommendation was expected to positively impact short -and long-term morbidity by avoiding radiation to the entire upper aerodigestive tract. It was also expected to positively impact prognosis compared to radiation alone.

**Head & Neck Case 8**

Short-term morbidity: better

Long-term morbidity: better

Prognosis: no change/unknown

A 55 year old woman came to MSK with an outside diagnosis of a 1.1 cm thyroid nodule, Bethesda category III, with suspicious molecular testing. The outside recommendation was for surgery (extent not specified). MSK did not meaningfully change the diagnosis. However, MSK recommendation was for observation instead of surgery due to the low risk nature of this diagnosis. The MSK change in treatment recommendation was expected to positively impact short- and long-term morbidity by avoiding surgery. The MSK change in treatment recommendation was not expected to impact prognosis.

**Head & Neck Case 9**

Short-term morbidity: better

Long-term morbidity: no change/unknown

Prognosis: no change/unknown

A 77 year old man came to MSK with an outside diagnosis of head and neck minor salivary gland adenoid cystic carcinoma of the hard palate, Stage IV. MSK did not meaningfully change the diagnosis. The outside recommendation was for surgery with free flap reconstruction, which MSK noted would likely have been followed by radiation once adverse risk factors were likely identified in the surgical specimen. MSK’s recommendation was for surgery with obturator instead of free flap reconstruction; in this case the free flap was deemed unnecessary to achieve optimal functional outcomes of speech and swallowing, thus offering no addition benefit to the patient. Surgery would most likely be followed by radiation. The MSK change in treatment recommendation was expected to positively impact short-term morbidity because of decreased operative time, decreased operative complications and faster recovery. The MSK change in treatment recommendation was not expected to impact long-term morbidity or prognosis.

**Head & Neck Case 10**

Short-term morbidity: better

Long-term morbidity: better

Prognosis: no change/unknown

A 67 year old man came to MSK with an outside diagnosis of poorly differentiated thyroid cancer metastatic to the lung, Stage IV. The outside recommendation was for total thyroidectomy. MSK did not meaningfully change the diagnosis, but performed MSK-IMPACT (integrated mutation profiling of actionable cancer targets) testing and identified a BRAF mutation amenable to drug therapy, which might help with future targeted treatments. MSK’s recommendation was for the first course of treatment to be hemithyroidectomy rather than total thyroidectomy to remove the gross primary site disease, followed by drug therapy. The MSK change in treatment recommendation was expected to positively impact short-and long-term morbidity because of decreased surgery and avoidance of the risk of bilateral recurrent laryngeal nerve injury. The MSK change in treatment recommendation was not expected to impact prognosis.

**Head & Neck Case 11**

Short-term morbidity: better

Long-term morbidity: better

Prognosis: no change/unknown

A 22 year old woman came to MSK with an outside diagnosis of a 2.0 cm papillary thyroid cancer. The outside recommendation was for total thyroidectomy, with possible transoral approach. MSK did not meaningfully change the diagnosis, but MSK’s recommendation was for hemithyroidectomy via traditional approach. The MSK change in treatment recommendation was expected to positively impact short- and long- term morbidity, not because of the change in surgical approach but rather due to change from total thyroidectomy to hemithyroidectomy. Hemithyroidectomy would reduce surgical risks and allow the patient the possibility of avoiding lifelong thyroid hormone replacement. The MSK change in treatment recommendation was not expected to impact prognosis.

**Head & Neck Case 12**

***Change in diagnosis**

Short-term morbidity: better

Long-term morbidity: better

Prognosis: no change/unknown

A 74 year old man came to MSK with an outside diagnosis of head and neck HPV-associated tonsil SCCA, Stage II. The outside recommendation was for chemoradiation. MSK changed the diagnosis upon review of the outside imaging, noting only ipsilateral instead of bilateral pathologic lymph nodes and downstaging to Stage I. Given this diagnostic change, MSK recommended reducing the extent of the radiation fields to only one side of the neck. Unrelated to this diagnostic change, MSK’s recommendation was to offer the patient participation in a clinical trial with a significantly lower dose of radiation (30 Gy instead of standard dose). The MSK change in treatment recommendation was expected to positively impact short- and long-term morbidity by decreasing the radiation dose. The MSK change in treatment recommendation was not expected to impact prognosis.

**Head & Neck Case 13**

Short-term morbidity: better

Long-term morbidity: better

Prognosis: no change/unknown

A 70 year old woman came to MSK with an outside diagnosis of a head and neck parotid gland benign Warthin's tumor. The outside recommendation was for surgery. MSK did not meaningfully change the diagnosis but did recommend changing the treatment plan from surgery to observation due to the low risk nature of this diagnosis. The MSK change in treatment recommendation was expected to impact short- and long-term morbidity by avoiding surgery. The MSK change in treatment recommendation was not expected to impact prognosis.

**Head & Neck Case 14**

Short-term morbidity: better

Long-term morbidity: better

Prognosis: no change/unknown

A 64 year old man came to MSK with an outside diagnosis of head and neck HPV-associated tonsil SCCA, Stage I. The outside recommendation was for surgical treatment. MSK did not meaningfully change the diagnosis. However, MSK’s recommendation was to offer the patient participation in a clinical trial with a significantly lower dose of radiation (30 Gy instead of standard dose). The MSK change in treatment recommendation was expected to positively impact short- and long-term morbidity by avoiding surgery and/or standard radiation dose. The MSK change in treatment recommendation was not expected to impact prognosis.

**Head & Neck Case 15**

Short-term morbidity: better

Long-term morbidity: better

Prognosis: no change/unknown

A 41 year old man came to MSK with an outside diagnosis of head and neck HPV-associated tonsil SCCA, Stage II. The outside recommendation was for chemoradiation. MSK did not meaningfully change the diagnosis. However, MSK’s recommendation was to offer the patient participation in a clinical trial with a significantly lower dose of radiation (30 Gy instead of standard dose). The MSK change in treatment recommendation was expected to positively impact short- and long-term morbidity by avoiding surgery and/or standard radiation dose. The MSK change in treatment recommendation was not expected to impact prognosis.

**Head & Neck Case 16**

Short-term morbidity: better

Long-term morbidity: better

Prognosis: no change/unknown

A 36 year old man came to MSK with an outside diagnosis of a 1.8 cm papillary thyroid cancer. The outside recommendation was for total thyroidectomy. MSK did not meaningfully change the diagnosis. However, MSK’s recommendation was for hemithyroidectomy. The MSK change in treatment recommendation was expected to positively impact short- and long-term morbidity due to decreased surgical risks and possibility of avoiding lifelong thyroid hormone replacement. The MSK change in treatment recommendation was not expected to impact prognosis.

**Head & Neck Case 17**

Short-term morbidity: worse

Long-term morbidity: better

Prognosis: no change/unknown

A 58 year old woman came to MSK with an outside diagnosis of head and neck sinonasal adenocarcinoma, Stage I. The outside recommendation was for endoscopic surgery followed by radiation. MSK changed the diagnosis, based on review of the outside pathology, to HPV-associated squamous cell carcinoma, with potential implications for prognosis and future treatments. MSK also changed the treatment recommendation to open surgical resection, due to the possibility of avoiding radiation if there were negative margins; this change was not based on the change in diagnosis. The MSK change in treatment recommendation was expected to lead to worse short-term morbidity due to open surgery but improved long-term morbidity due to the avoidance of radiation, with no impact on prognosis.

**Lung Case 1**

Short-term morbidity: better

Long-term morbidity: no change/unknown

Prognosis: better

A 76 year old man came to MSK with an outside diagnosis of lung cancer, Stage IV. The outside recommendation was for chemotherapy. MSK did not meaningfully change the diagnosis but changed the treatment recommendation to chemotherapy and immunotherapy. The MSK change in treatment recommendation was expected to improve prognosis due to the addition of immunotherapy and to improve short-term morbidity by more effectively reducing symptoms related to the disease. The MSK change in treatment recommendation had uncertain expected impact on long-term morbidity.

**Lung Case 2**

Short-term morbidity: worse

Long-term morbidity: better

Prognosis: better

A 78 year old woman came to MSK with an outside diagnosis of lung cancer, Stage IIIB. The outside recommendation was for chemotherapy. MSK did not meaningfully change the diagnosis but did conduct additional brain imaging to complete staging workup and rule out metastases. MSK also noted that the outside treatment recommendation was not guideline concordant: This is a potentially curable disease state, and the outside recommendation was not a curative regimen. MSK therefore recommended chemoradiation and adjuvant durvalumab, as consistent with guidelines for treating Stage IIIB lung cancer. The MSK change in treatment recommendation was expected to positively impact prognosis and long-term morbidity, and possibly to worsen short-term morbidity.

**Lung Case 3**

Short-term morbidity: better

Long-term morbidity: no change/unknown

Prognosis: no change/unknown

A 63 year old man came to MSK with an outside diagnosis of lung adenocarcinoma, Stage IV with leptomeningeal involvement. The outside recommendation was for chemotherapy with immunotherapy and whole brain radiation. MSK did not meaningfully change the diagnosis but did change the treatment recommendation to chemotherapy and immunotherapy, while waiting to start or potentially avoiding whole brain radiation. Whole brain radiation is appropriate when there are central nervous symptoms, and/or after the lung disease has responded to treatments; but in this case, since there were no central nervous system symptoms, whole brain radiation would delay treating symptoms caused by the lung disease. Furthermore, whole brain radiation does not impact survival. The MSK change in treatment recommendation was expected to improve short-term morbidity due to decreased toxicity by avoiding whole brain radiation. The MSK change in treatment recommendation was not expected to impact prognosis or long-term morbidity.

**Lung Case 4**

***Change in diagnosis**

Short-term morbidity: better

Long-term morbidity: better

Prognosis: better

A 67 year old woman came to MSK with an outside diagnosis of lung cancer, Stage IV. The outside treatment recommendation was for afatinib, a tyrosine kinase inhibitor. MSK changed the diagnosis after conducting a repeat biopsy with enough material to determine the type of EGFR (epidermal growth factor receptor) mutation, identifying the precise exon insertion. Based on this change in diagnosis, MSK changed the treatment recommendation; while some EGFR exon insertions are sensitive to tyrosine kinase inhibitors, this patient's tumor was not -- therefore requiring chemotherapy. The MSK change in treatment recommendation was expected to improve prognosis due to avoidance of an ineffective treatment. The MSK change in treatment recommendation was also expected to improve short- and long-term morbidity due to using an appropriate targeted therapy.

**Lung Case 5**

Short-term morbidity: better

Long-term morbidity: better

Prognosis: no change/unknown

A 63 year old woman with advanced COPD with poor pulmonary reserve on home oxygen came to MSK with an outside diagnosis of lung adenocarcinoma, Stage IA. The outside recommendation was for surgery. MSK did not meaningfully change the diagnosis but did change the treatment recommendation to radiation. The MSK change in treatment recommendation was expected to improve short- and long-term morbidity due to non-surgical therapy. With poor pulmonary reserve, surgical management would have resulted in a higher risk of post-operative complications and worse quality of life. The MSK change in treatment recommendation was not expected to impact prognosis.

**Lung Case 6**

Short-term morbidity: better

Long-term morbidity: no change/unknown

Prognosis: no change/unknown

A 57 year old man came to MSK with an outside diagnosis of small cell lung cancer. The outside treatment recommendation was for induction chemotherapy and possible surgery. MSK did not meaningfully change the diagnosis. However, the outside treatment recommendation was not guideline concordant, so MSK instead recommended guideline-concordant chemoradiation. The MSK change in treatment recommendation was expected to positively impact short-term morbidity due to avoidance of surgery. The MSK change in treatment recommendation was not expected to impact long-term morbidity or prognosis.

**Lung Case 7**

***Change in diagnosis**

Short-term morbidity: better

Long-term morbidity: no change/unknown

Prognosis: no change/unknown

A 70 year old woman came to MSK with an outside diagnosis of a lung nodule on imaging concerning for lung cancer. The outside recommendation was for surgical lobectomy, despite not having a cancer diagnosis. The lobectomy would diagnose and treat the nodule in one procedure, should it prove cancerous. MSK completed and refined the diagnosis by recommending a biopsy via needle– this diagnostic procedure was less invasive than surgery and showed the nodule to be benign. Given this finding, MSK recommended no treatment. The MSK change in management to refine the diagnosis was expected to positively impact short-term morbidity because the lesion turned out to be benign, allowing the patient to avoid surgical lobectomy. The MSK change in treatment recommendation was not expected to impact long-term morbidity or prognosis.

**Lung Case 8**

Short-term morbidity: worse

Long-term morbidity: better

Prognosis: better

A 76 year old man came to MSK with an outside diagnosis of carcinoid lung cancer, Stage III.
The outside recommendation was for chemotherapy only. MSK did not meaningfully change the diagnosis. However, MSK changed the treatment recommendation, as the outside plan was not guideline concordant: This is a potentially curable disease state, and the outside recommendation was not a curative regimen. Instead, MSK recommended guideline-concordant chemoradiation. The MSK change in treatment recommendation was expected to positively impact prognosis with the potential for cure. It was also expected to positively impact long-term morbidity due to potential for cure, with worse short-term morbidity due to the addition of radiation.

**Lung Case 9**

***Change in diagnosis**

Short-term morbidity: better

Long-term morbidity: no change/unknown

Prognosis: better

A 62 year old man came to MSK with an outside diagnosis of a spiculated lung lesion concerning for lung cancer. The outside recommendation was for surgical lobectomy, in order to diagnose and treat the cancer in one procedure, should it prove cancerous. MSK recommended completing the diagnosis via needle biopsy rather than surgical lobectomy– the results of which showed small cell lung cancer for which primary surgery is not recommended. The MSK change in treatment recommendation was expected to positively impact short-term morbidity by avoiding unnecessary surgical lobectomy. It was also expected to positively impact prognosis, because the diagnosis turned out to be cancer for which initial surgical treatments are not recommended. The MSK recommendation was not expected to impact long-term morbidity.

**Lung Case 10**

Short-term morbidity: worse

Long-term morbidity: better

Prognosis: better

A 60 year old man came to MSK with an outside diagnosis of locally advanced lung squamous cell lung cancer. The outside recommendation was for chemoradiation followed by adjuvant immunotherapy. MSK did not meaningfully change the diagnosis. However, MSK changed the treatment recommendation by offering the patient participation in a neoadjuvant clinical trial with chemotherapy followed by surgery and then immunotherapy. The MSK change in treatment recommendation was expected to positively impact prognosis and long-morbidity due to the addition of surgery, which, when feasible, may provide better oncologic outcomes, especially when combined with adjuvant immunotherapy. The MSK change in treatment recommendation was expected to lead to worse short-term morbidity due to the addition of surgery.

**Lung Case 11**

Short-term morbidity: worse

Long-term morbidity: better

Prognosis: better

An 89 year old woman came to MSK with an outside diagnosis of non-small cell lung cancer, Stage IV. The outside recommendation was for palliation with immunotherapy and radiation. MSK did not meaningfully change the diagnosis. However, MSK’s treatment recommendation was for immunotherapy and chemotherapy, for better palliation. The MSK change in treatment recommendation was expected to positively impact prognosis and long-morbidity due to improved palliation, with worse short-term morbidity due to receiving chemotherapy instead of radiation.

**Myeloma Case 1**

***Change in diagnosis**

Short-term morbidity: better

Long-term morbidity: no change/unknown

Prognosis: no change/unknown

A 69 year old woman came to MSK with an outside diagnosis of active multiple myeloma after having been followed for smoldering multiple myeloma. The outside recommendation was to start systemic therapy for active myeloma. MSK changed the diagnosis back to smoldering multiple myeloma (intermediate-risk), based on review of outside studies. Based on the change in diagnosis, MSK changed the treatment recommendation to continued observation, as there was no clear evidence of progression to active multiple myeloma requiring therapy. The MSK change in treatment recommendation was expected to positively impact short-term morbidity due to avoiding systemic therapy. It was unknown whether the MSK change in treatment recommendation would impact long-morbidity or prognosis. Because the majority of patients with smoldering myeloma will eventually need systemic therapy, we can only be confident about improving short-term morbidity at present.

**Myeloma Case 2**

***Change in diagnosis**

Short-term morbidity: better

Long-term morbidity: better

Prognosis: better

A 41 year old man came to MSK with an outside diagnosis of active multiple myeloma, based in part on interpretation of bone pain as active disease. The outside recommendation was to start systemic therapy for active myeloma. MSK changed the diagnosis to MGUS (monoclonal gammopathy of unknown significance), based on review of outside studies; MSK also found the patient to have neuropathy and joint pain rather than bone pain. Based on the change in diagnosis, MSK changed the treatment recommendation to continued observation, because there was no clear evidence of progression to active multiple myeloma requiring therapy. The MSK change in treatment recommendation was expected to positively impact short- and long-term morbidity, as the large majority of patients with MGUS will never require systemic therapy. The MSK change in treatment recommendation was also expected to positively impact prognosis by avoiding potentially toxic (or even life-threatening, albeit rarely) therapies that, most likely, would never be needed.

**Myeloma Case 3**

Short-term morbidity: better

Long-term morbidity: no change/unknown

Prognosis: no change/unknown

A 63 year old man came to MSK with an outside diagnosis of intermediate-high-risk smoldering myeloma. The outside recommendation was to start systemic therapy on a clinical trial. MSK did not meaningfully change the diagnosis. However, MSK changed the treatment recommendation to continued observation, as there was no clear evidence of progression to active multiple myeloma requiring therapy. The MSK change in treatment recommendation was expected to positively impact short-term morbidity due to avoiding systemic therapy. It was unknown whether the MSK change in treatment recommendation would impact long-term morbidity or prognosis; it is possible that long term morbidity and prognosis could be worse without participation in the clinical trial (if on drug treatment for the trial) but this is not currently known and is not the standard of care. Because the majority of patients with smoldering myeloma will eventually need systemic therapy, we can only be confident about improving short-term morbidity at present.

**Myeloma Case 4**

***Change in diagnosis**

Short-term morbidity: better

Long-term morbidity: no change/unknown

Prognosis: no change/unknown

A 71 year old man came to MSK with an outside diagnosis of smoldering multiple myeloma, with a retroperitoneal mass interpreted as end-organ damage attributable to plasma cell disorder. The outside recommendation was to start systemic therapy based on the retroperitoneal finding. MSK did not change the diagnosis of smoldering myeloma but did change the diagnosis of the retroperitoneal mass to retroperitoneal fibrosis unrelated to plasma cell disorder, rather than end-organ-damage due to a plasma cell disorder, based on review of outside radiology. MSK then changed the treatment recommendation to observation for the smoldering myeloma, and prednisone for the retroperitoneal fibrosis. The MSK change in treatment recommendation was expected to positively impact short-term morbidity due to avoiding systemic therapy. It was unknown whether the MSK change in treatment recommendation would impact long-morbidity or prognosis. Because the majority of patients with smoldering myeloma will eventually need systemic therapy, we can only be confident about improving short-term morbidity at present.

**Myeloma Case 5**

Short-term morbidity: better

Long-term morbidity: no change/unknown

Prognosis: no change/unknown

A 47 year old woman came to MSK with an outside diagnosis of intermediate-risk smoldering multiple myeloma. The outside recommendation was to start systemic therapy but not on a clinical trial, which is currently the recommended and preferred approach for therapy initiation in patients with smoldering myeloma according to guidelines. MSK did not change the diagnosis. However, MSK changed the treatment recommendation to observation, as there was no clear evidence of progression to active multiple myeloma requiring therapy, and it would only be appropriate to treat smoldering myeloma in a clinical trial setting. The MSK change in treatment recommendation was expected to positively impact short-term morbidity due to avoiding systemic therapy. It was unknown whether the MSK change in treatment recommendation would impact long-morbidity or prognosis. Because the majority of patients with smoldering myeloma will eventually need systemic therapy, we can only be confident about improving short-term morbidity at present.

**Myeloma Case 6**

Short-term morbidity: no change/unknown

Long-term morbidity: better

Prognosis: no change/unknown

A 68 year old man came to MSK with an outside diagnosis of multifocal plasmacytomas. The outside recommendation was to start systemic therapy, which is standard of care for two or more plasmacytomas. MSK did not change the diagnosis, but review of outside radiology showed that the patient had only two plasmacytomas, and that they were in close proximity to each other. MSK therefore changed the treatment recommendation to radiation therapy: Given their close proximity, both plasmacytomas could be treated within a single radiation field. The MSK recommendation was expected to positively impact long-term morbidity due to avoiding systemic therapy. The MSK change in treatment recommendation was not expected to impact short-term morbidity, because the patient would still have morbidity from radiation; nor was it expected to impact prognosis due to similar outcomes between systemic therapy and radiation.

**Myeloma Case 7**

***Change in diagnosis**

Short-term morbidity: better

Long-term morbidity: no change/unknown

Prognosis: no change/unknown

An 84 year old man came to MSK with an outside diagnosis of active multiple myeloma after having been followed for smoldering multiple myeloma. The outside recommendation was to start systemic therapy for active myeloma. MSK changed the diagnosis back to smoldering multiple myeloma, based on review of outside studies. Based on the change in diagnosis, MSK changed the treatment recommendation to continued observation, as there was no clear evidence of progression to active multiple myeloma requiring therapy. The MSK change in treatment recommendation was expected to positively impact short-term morbidity due to avoiding systemic therapy. It was unknown whether the MSK change in treatment recommendation would impact long-term morbidity or prognosis. Because the majority of patients with smoldering myeloma will eventually need systemic therapy, we can only be confident about improving short-term morbidity at present.
